# Orientation-dependent structural and photocatalytic properties of LaCoO_3_ epitaxial nano-thin films

**DOI:** 10.1098/rsos.171376

**Published:** 2018-02-14

**Authors:** Yan-ping Zhang, Hai-feng Liu, Hai-long Hu, Rui-shi Xie, Guo-hua Ma, Ji-chuan Huo, Hai-bin Wang

**Affiliations:** 1 School of Materials Science and Engineering, Southwest University of Science and Technology, Mianyang 621010, China; 2 Analytical and Testing Center, Southwest University of Science and Technology, Mianyang 621010, China; 3 School of Chemistry and Chemical Engineering, Mianyang Normal University, Mianyang 621010, China

**Keywords:** LaCoO_3_, orientation, photocatalysis, epitaxial film, strain

## Abstract

LaCoO_3_ epitaxial films were grown on (100), (110) and (111) oriented LaAlO_3_ substrates by the polymer-assisted deposition method. Crystal structure measurement and cross-section observation indicate that all the LaCoO_3_ films are epitaxially grown in accordance with the orientation of LaAlO_3_ substrates, with biaxial compressive strain in the *ab* plane. Owing to the different strain directions of CoO_6_ octahedron, the mean Co–O bond length increases by different amounts in (100), (110) and (111) oriented films compared with that of bulk LaCoO_3_, and the (100) oriented LaCoO_3_ has the largest increase. Photocatalytic degradation of methyl orange indicates that the order of photocatalytic activity of the three oriented films is (100) > (111) > (110). Combined with analysis of electronic nature and band structure for LaCoO_3_ films, it is found that the change of the photocatalytic activity is closely related to the crystal field splitting energy of Co^3+^ and Co–O binding energy. The increase in the mean Co–O bond length will decrease the crystal field splitting energy of Co^3+^ and Co–O binding energy and further reduce the value of band gap energy, thus improving the photocatalytic activity. This may also provide a clue for expanding the visible-light-induced photocatalytic application of LaCoO_3_.

## 1. Introduction

In recent years, with the increasingly prominent problem of environmental pollution, photocatalytic materials based on perovskite or perovskite-derived structures have become the focus of scholars' research and attention due to their excellent performance for degradation of persistent organic pollutants [1–3]. Compared with the traditional TiO_2_ photocatalyst, ABO_3_ perovskite structures have advantages of narrower band gap, wider wavelength range of light absorption, higher utilization rate of sunlight, lower electron–hole recombination rate, higher quantum efficiency and so on [4–6]. Among them, as a significant member of perovskite-type cobalt oxides, nanoscale LaCoO_3_ (LCO) has not only the spin state change and low-temperature ferromagnetic behaviour [7–9], but also good photocatalytic activity. Besides, the octahedral crystal field of CoO_6_ octahedron always splits the fivefold 3*d* orbital of Co^3+^ into higher doublet *e*_g_ orbital and lower triplet *t*_2 g_ orbital when disrupted by different effects such as doping and chemical or external pressure, thus making electrons transfer from the *t*_2 g_ level to the *e*_g_ level easily, which is conducive to the generation of electron–hole pairs and the improvement of photocatalytic activity [10]. Based on this feature, LCO is considered as a promising photocatalytic material. In addition, recent experiments have indicated that LCO or doped LCO nano-thin films, especially epitaxial films with different crystal orientations, show novel and different magnetic and electrical properties and reduction activity [11–15]. However, as far as we know, there is no relevant report on the photocatalytic properties of LCO epitaxial nano-thin films with different crystal orientations. Thus, further studies are necessary to explore whether different photocatalytic properties exist in LCO epitaxial films with different crystal orientations. This may also help to understand the nature of the photocatalytic activity in LCO films.

Thus, in this paper, LCO nano-thin epitaxial films were grown on (100), (110) and (111) oriented LaAlO_3_ (LAO) substrates, by a simple and cost-efficient polymer-assisted deposition (PAD) method, instead of one of the many vacuum techniques [15–19] which usually require high-cost equipment and strict deposition conditions. In the PAD process, an aqueous solution of metal precursors is mixed well with a soluble polymer which actively binds and encapsulates the metal ions and helps to both prevent hydrolysis reaction and distribute them uniformly in the solution. Therefore, it has been proved as a new and effective method to grow crack-free and relatively thick epitaxial films with desired chemical composition accordingly [20–22]. Based on characteristics for the morphology, crystal structure distortion and optical absorption properties, the orientation-dependent epitaxial nature and photocatalytic activity of the LCO epitaxial films were investigated. Furthermore, to compare the orientation-dependent photocatalytic performance of the LCO epitaxial films, further experiments of photocatalytic degradation of methyl orange were carried out. The results will provide a striking proof to reveal the interrelationship between orientation-dependent structural and photocatalytic properties of the LCO nano-thin epitaxial films.

## 2. Experimental

### 2.1. Preparation of the LaCoO_3_ epitaxial films

LCO epitaxial films with different crystal orientations were prepared by the PAD method, in which polyethyleneimine (PEI) with a molecular weight of 70 000 was used as a binding agent to the metal ions. The precursor solution was prepared as follows. First, high-purity (greater than 99.99%) metal salts La(NO_3_)_3_·*n*H_2_O (0.5 mmol) and Co(NO_3_)_2_·6H_2_O (0.5 mmol) were mixed and dissolved in 5 ml deionized water, and then ethylenediaminetetraacetic acid (1 mmol) was added. After 30 min chelating reaction, PEI (0.2922 g) was dissolved in 10 ml deionized water and added to the above solution slowly using a dropper. The mixed solution was stirred by a magnetic stirring apparatus for 12 h at room temperature, and then kept stirred in a 60°C oil bath until approximately 5 ml solution remained.

Subsequently, the precursor solution was deposited on treated single-crystal (100), (110) and (111) oriented LAO substrates (10 × 5 × 0.5 mm) at 4000 r.p.m. over 30 s by a spin coating technique. It should be mentioned that all of the used substrates were double-sided polished and produced in a class 1000 clean room by Hefei Kejing Materials Technology Co. Ltd, having a highly clean surface and no further pre-treatment. Then the coated substrates were heated up at a rate of 1°C min^−1^ from room temperature to 700°C to make sure the water evaporated and polymers burned up to avoid the formation of voids in the bulk of the films. The samples were then rapidly heated (10°C min^−1^) to a temperature of 900°C. After 2 h heat treatment, the films were cooled down to room temperature at 1°C min^−1^.

### 2.2. Characterizations

The surface morphology and thickness of the film samples were observed with a field emission scanning electron microscope (FE-SEM, Carl Zeiss Ultra 55). The structural and epitaxial characterization of the films including *θ*/2*θ* symmetric scan, *ω*-scans (rocking curve) as well as in-plane *φ*-scan were performed via an X-ray diffraction (XRD) (Rigaku D/max TTRШ and PANalytical X'Pert PRO) with Cu *K*_α_ radiation. To further identify the epitaxial growth of the films, the cross section and lattice fringe phase of the LCO films on LAO substrates were investigated by a high-resolution transmission electron microscope (TEM, FEI Tecnai G2 F20 S-Twin). In addition, the change of Co–O bond lengths and vibrational modes were measured by a confocal microprobe Raman spectrometer (Renishaw InVia) ranging from 130 to 1000 cm^−1^ at room temperature. The electronic natures of Co^3+^ ions for different oriented LCO films were investigated using an X-ray photoelectron spectroscopy (XPS) instrument (Thermo Fisher Scientific, ESCALAB 250Xi) with a monochromatized Al-*K*_α_ source (*hν* = 1486.6 eV) and the binding energy was calibrated by C1*s* as reference energy (C1*s* = 284.8 eV). The light absorption range and intensity of the LCO epitaxial films were analysed by a UV–visible–near infrared spectrometer (UV–Vis–NIR, Shimadzu SolidSpec-3700).

### 2.3. Photocatalytic degradation experiments

The photocatalytic degradation experiments were carried out as follows. Each prepared LCO film was placed in 50 ml solution of methyl orange with a concentration of 5 mg l^−1^ and with the coating face upward. A 300 W high-pressure mercury lamp (with dominant wavelength 365–400 nm and a part of visible light) was hung at 20 cm above the solution which was subjected to mechanical agitation. A sample was taken every hour and the absorbance measured at the maximum absorption wavelength of the dye (*λ* = 465 nm) by a UV–Vis–NIR (Shimadzu UV-3150). While the decolorization (*D*_e_) was calculated according to the absorbance values of the solution before and after the reaction, *D*_e_ = [(*A*_0 _− *A*)/*A*_0_] × 100%, where *A*_0_ is the absorbance before the reaction and *A* the absorbance after the reaction.

## 3. Results and discussion

### 3.1. Morphological and structural characterization

**Figure 1.**
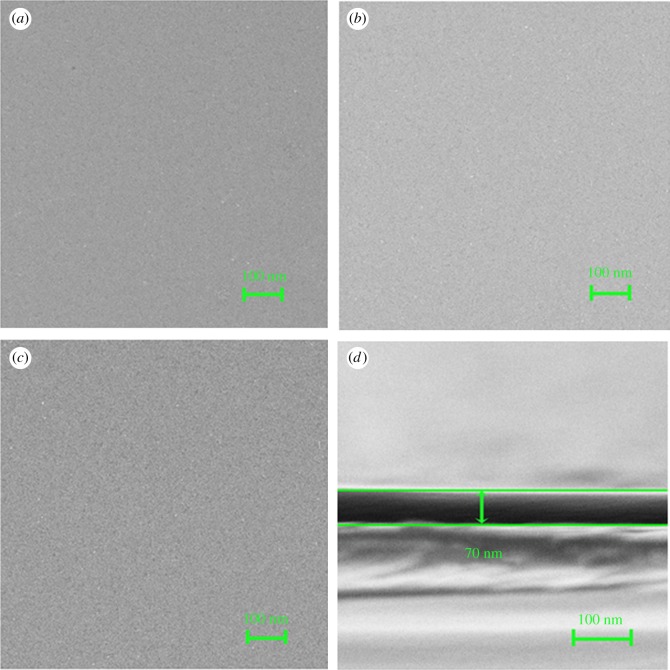
The top-view FE-SEM and cross-section images of the LCO films: (*a*), (*b*) and (*c*) show the surface morphology of (100), (110) and (111) oriented LCO films coated at 4000 r.p.m., respectively; (*d*) shows the cross-sectional image of (100) oriented LCO film coated at 4000 r.p.m.

**Figure 2.**
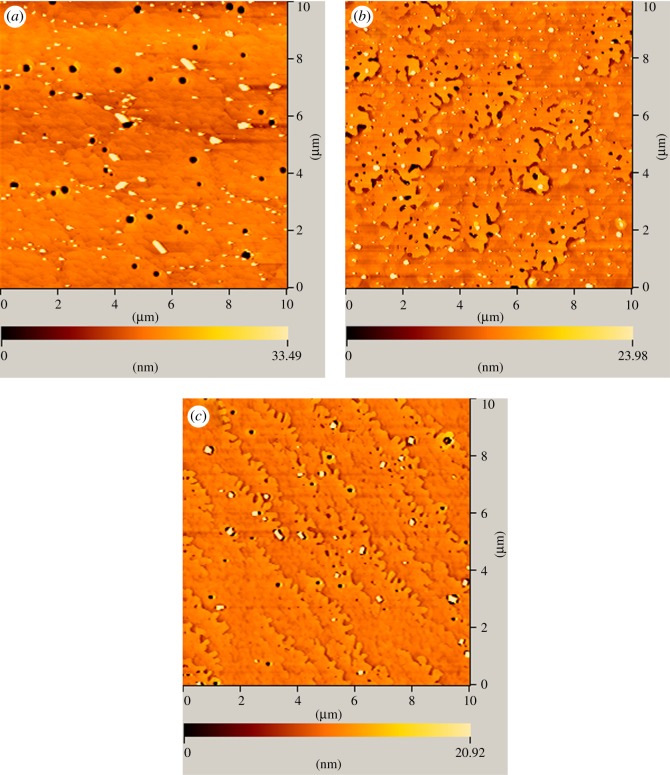
The AFM images of LCO epitaxial films on (*a*) (100), (*b*) (110) and (*c*) (111) LAO substrates.

Figure 1 shows the top-view FE-SEM and cross-sectional images of the LCO epitaxial films. From the surface morphologies of (100), (110) and (111) oriented LCO films coated at 4000 r.p.m., as shown in figure 1*a*–*c*, it can be seen that all the films are homogeneous and dense. From figure 1*d* corresponding to the cross-sectional area of LCO film grown on (100) LAO substrate, the film thickness can be measured to be approximately 70 nm, consistent with those of the other two oriented films. Energy-dispersive X-ray analysis (not shown here) shows only the characteristic peaks of La, Al, Co and O; no absorption peaks of other impurity elements are detected. In addition, the atomic force microscopy (AFM) images of (100), (110) and (111) oriented LCO films are shown in figure 2*a*–*c*. It shows that the films are uniform over the scanned surface (10 × 10 μm) and the surface roughness is less than 5 nm. However, some tiny pores exist which may be due to the volatilization of PEI during heat treatment.

**Figure 3.**
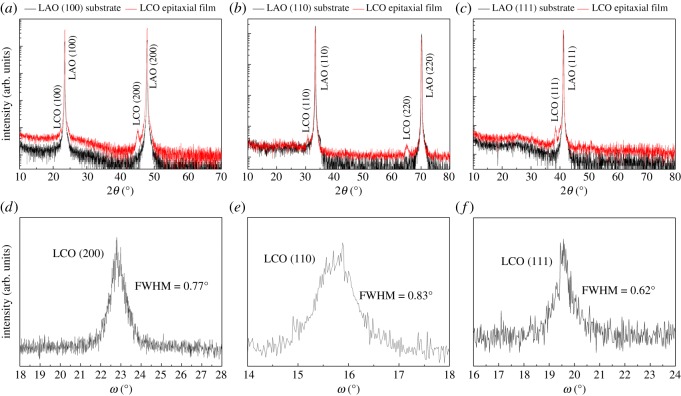
The typical XRD patterns of the LCO epitaxial films: (*a*), (*b*) and (*c*) are the *θ*/2*θ* symmetric scans of the films grown on (100), (110) and (111) LAO substrates, respectively; (*d*), (*e*) and (*f*) are the corresponding *ω*-scans (rocking curves) of the films, respectively.

**Figure 4.**
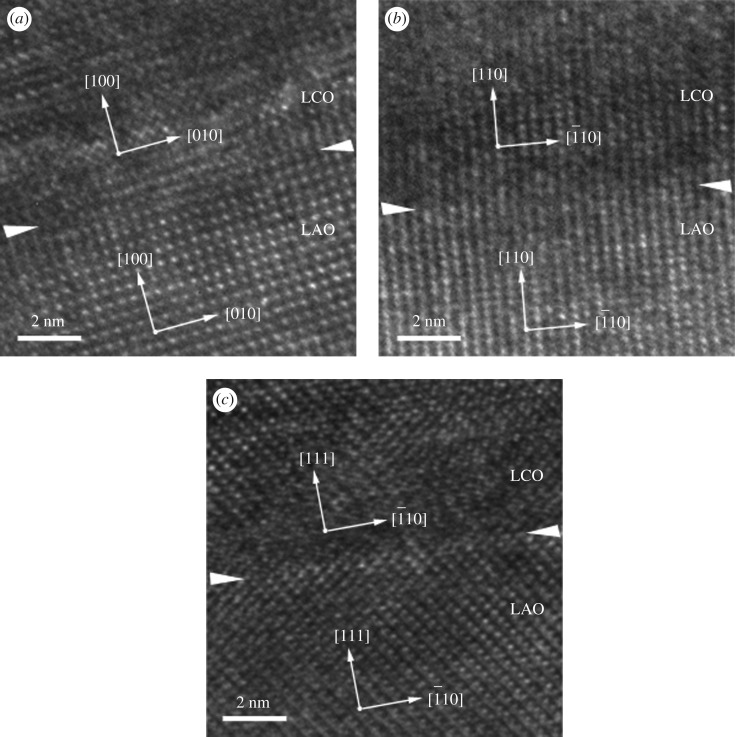
The high-resolution TEM images from the interface between LCO films on (*a*) (100), (*b*) (110) and (*c*) (111) LAO substrates.

The typical XRD patterns of the LCO epitaxial films are shown in figure 3, in which figure 3*a*–*c* shows the *θ*/2*θ* symmetric scans of the films grown on (100), (110) and (111) LAO substrates, and figure 3*d*–*f* shows the corresponding *ω*-scans (rocking curves) of the LCO films, respectively. All films show clear diffraction peaks accompanied by the diffraction peaks of differently oriented LAO substrates, and no diffraction peaks of impurity phase or other orientation are observed. It should be mentioned that LCO powder and LCO polycrystalline film deposited on (100) oriented Si were also prepared by the PAD method in the experiment. The corresponding XRD results (not shown here) prove that both of them are pure phase. Therefore, it can be concluded that the LCO epitaxial films prepared by the PAD method are of high purity. Combined with the *ω*-scans of the films, it illustrates that each oriented LCO film has a good out-of-plane orientation. Here, the width of the *ω*-scans for the (100), (110) and (111) oriented LAO substrates are 0.06°, 0.05° and 0.03°, respectively. In addition, the asymmetric in-plane *φ*-scans (not shown here) of films and substrates indicate that the in-plane symmetry of each film is consistent with that of the corresponding substrate. These results demonstrate that each LCO film shows epitaxial growth in accordance with the orientation of the LAO substrate. Moreover, the epitaxial nature of the LCO film is also verified by the high-resolution TEM analysis. Figure 4*a*–*c* presents the high-resolution TEM images from the interface between LCO and (100), (110), (111) LAO, respectively. As can be seen, the films are orderly grown in a layered form on the corresponding substrate. It indicates the high crystallinity of the films and also confirms the epitaxial growth of LCO films on the three different oriented LAO substrates, which is consistent with the XRD results.

Based on the XRD results, the out-of-plane (perpendicular to the film surface) interplanar spacing of (100), (110) and (111) oriented LCO films are calculated and listed in table 1. As is known, bulk LCO can be regarded as a pseudocubic perovskite structure deformed in (111) direction with a lattice parameter of *a*_bulk_ ≈ 3.805 Å [21,23]. Owing to the smaller lattice constant of LAO substrate (*a*_sub_ ≈ 3.791 Å), all the LCO films grown on LAO substrates are subjected to biaxial compressive strain in the *ab* plane (parallel to the film surface), resulting in the stretching effect perpendicular to the film surface and the distortion of CoO_6_ regular octahedrons [24]. As is known, the ideal values of the out-of-plane interplanar spacing for the (100), (110) and (111) planes of LCO bulk are about 3.80, 2.69 and 2.20 Å, respectively [12]. Thus, the out-of-plane strain, shown in table 1, can be evaluated through the formula *ε*_out_ = (*c*_film _− *c*_bulk_)/*c*_bulk_, where *c*_film_ and *c*_bulk_ are the out-of-plane interplanar spacing of LCO epitaxial film and LCO bulk, respectively. For (100) oriented LCO film, the out-of-plane lattice parameter is about 4.014 Å, larger than the value in Herklotz's work [25]. This difference may be attributed to the relaxation of the biaxial compressive strain. Because of the lattice relaxation effect in epitaxial thin films, the biaxial compressive strain may be relaxed and the average *c*-axis constant will tend to the value of bulk LCO (3.805 Å) when the LCO film becomes thicker. In Herklotz's work, the thickness of LCO films is about 100 nm, while that of films in our work is thinner (about 70 nm). Thus, the thinner LCO film suffers greater compressive strain and has greater stretching effect perpendicular to the film surface, corresponding to a large out-of-plane lattice parameter. Furthermore, from table 1, it is evident that the different oriented films are all subjected to out-of-plane tensile strain, although the value of *ε*_out_ is very close. However, the values of lattice mismatch for LCO epitaxial films with different orientations, (*a*_bulk _− *a*_sub_)/*a*_sub_, remain constant (about 0.37%). It means that the out-of-plane tensile stress (*σ*) of the three oriented LCO films has the same value. Thus, the different out-of-plane strain of the three oriented LCO films may be attributed to the different elastic modulus defined as *E* = *σ*/*ε.* For perovskite structures, it should be noted that the (111) plane is the close-packed plane of atoms with the maximum elastic modulus along the 〈111〉 crystallographic direction [26,27]. The largest elastic modulus and the smallest lattice disorder in (111) oriented LCO film can lead to the smallest lattice disorder and out-of-plane strain under the same stress. Compared with the elastic modulus of (111) oriented LCO films, it is smaller along the 〈110〉 crystallographic direction for (110) oriented LCO film, while (100) oriented LCO film corresponds to the minimum elastic modulus. Therefore, the largest out-of-plane strain exists in the (100) oriented LCO epitaxial film, resulting in elongation of the CoO_6_ octahedron along the O–Co–O bond perpendicular to the film surface [27,28].

**Table 1.** The out-of-plane interplanar spacing (*c*_film_) and tensile strain (*ε*_out_) of the (100), (110) and (111) oriented LCO epitaxial films at room temperature.

substrate	lattice plane	*c*_film_ (Å)	*ε*_out_ (%)
(100) LAO	(100)	4.014	+5.63
(110) LAO	(110)	2.837	+5.46
(111) LAO	(111)	2.318	+5.36

**Figure 5.**
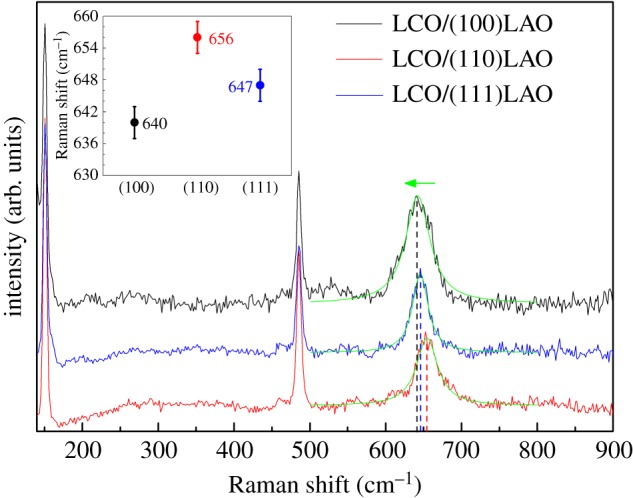
Raman spectra of the LCO epitaxial films on (100), (110) and (111) LAO substrates; the inset shows the peak positions of the Co–O stretching vibration located at approximately 650 cm^−1^.

To investigate the structure distortion of the LCO epitaxial films with different orientations, Raman spectra were obtained at room temperature (figure 5). Besides several Raman bands of LAO substrate, the broad mode of LCO film located at approximately 650 cm^−1^ belongs to the Co–O stretching vibration [29,30]. The change of this Raman peak has a great relationship with the change of mean Co–O bond length [31] which may result from the epitaxial strain-induced structure distortion of CoO_6_ regular octahedrons [30]. Thus, the increase in the intensity of the 650 cm^−1^ band and the shift of the peak correlate with evidence of stretching effect created by a perovskite deformation, for which the stretching effect stabilizes the state with longer mean Co–O bond length. Therefore, this downshift corresponds to the softening of the Co–O bands and larger mean distances of Co–O bonds, which is consistent with the larger out-of-plane interplanar spacing for LCO epitaxial film grown on LAO. As shown in figure 5, with the substrate orientation changing from (110), (111) to (100), the position of Co–O stretching vibration shifts to lower wavenumbers, which may suggest an increase in the mean Co–O bond length.

**Figure 6.**
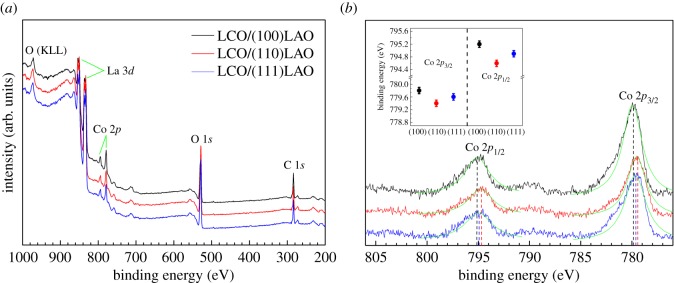
X-ray photoelectron spectra of LCO epitaxial films on (100), (110) and (111) LAO substrates: (*a*) the survey scan for LCO films; (*b*) the spectra of Co 2*p* core levels; the inset shows the binding energies of Co 2*p*_1/2_ and Co 2*p*_3/2_.

In order to further study the orientation-dependent Co–O bond change and Co^3+^ crystal field splitting nature of the LCO epitaxial films, XPS analyses were conducted as shown in figure 6, and the binding energies of Co 2*p* and O 1*s* core levels are summarized in table 2. Figure 6*a* shows the high-sensitivity survey scan of different oriented LCO films, which contains the peaks of C, O, La and Co elements. Figure 6*b* displays the high-resolution XPS spectra of Co 2*p* core levels for different oriented LCO films. It can be seen that the Co 2*p* spectrum shows no signal at 778.3 eV, indicating that there is no detectable free Co metal in the sample [15]. The Co 2*p*_3/2_ peaks of as-grown LCO films show a small shift towards lower binding energy than the unstressed value (780.0 eV) in previous studies [32–34], which may be associated with the in-plane compressive strain of LCO film grown on the LAO substrate. The existence of compression effect can lead to an increase in Co^3*d*^–O^2*p*^ hybridization [21]. These differences in the binding energy of different orientations may be related to the different degree of hybridization. Besides, the increase of mean Co–O bond length results in the enhancement of hybridization, while the stronger hybridization corresponds to higher binding energy [24]. This is consistent with the Raman analysis.

**Table 2.** XPS binding energies (eV) of photoelectron peaks of the synthesized (100), (110) and (111) oriented LCO epitaxial films.

sample	Co 2*p*_1/2_	Co 2*p*_3/2_	O 1*s*
LCO/(100)LAO	795.17	779.82	531.42
			528.77
LCO/(110)LAO	794.57	779.37	532.12
			529.02
LCO/(111)LAO	794.92	779.62	530.17
			528.82

### 3.2. Photocatalytic characterization

**Figure 7.**
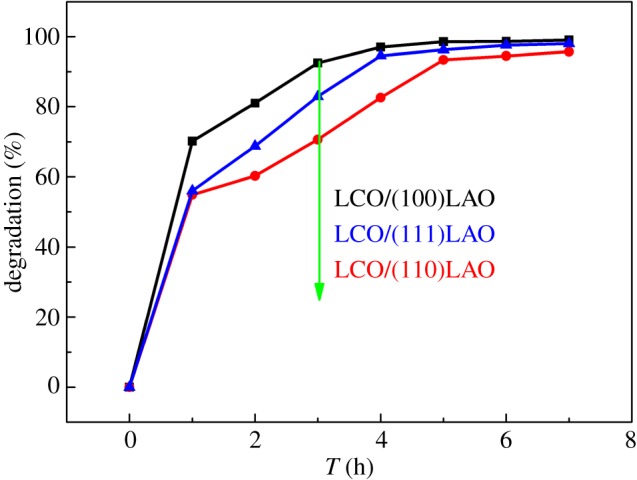
Photocatalytic degradation of methyl orange by LCO epitaxial films on (100), (110) and (111) LAO substrates.

**Figure 8.**
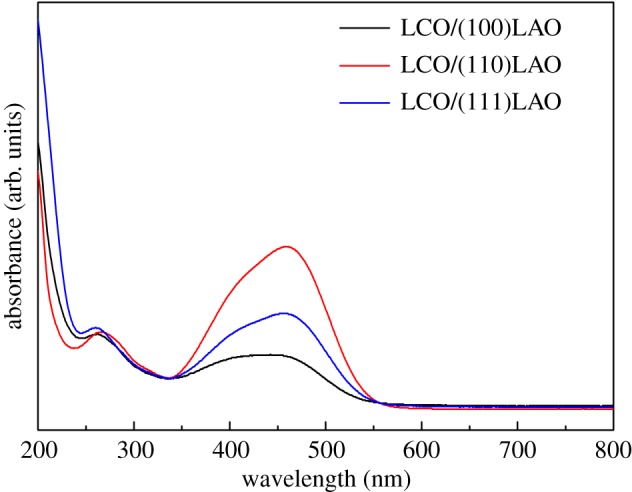
The UV–visible absorption spectra of methyl orange solution after 3 h of reaction using LCO epitaxial films on (100), (110) and (111) LAO substrates.

Figure 7 presents photocatalytic degradation of methyl orange solution by (100), (110) and (111) oriented LCO epitaxial films. The photocatalytic contribution from different oriented LAO substrate has been measured separately and subtracted from the photocatalytic degradation data. It can be found from the degradation curves that all the films exhibit strong photocatalytic performance. The degradation rate of the methyl orange solution is fastest in the first 2 h, and then became gentle after 5 h. Nevertheless, the three oriented LCO films show different photocatalytic degradation behaviours. The (100) oriented LCO film shows the best photocatalytic activity with 99.06% degradation of methyl orange solution in 7 h. For the (110) oriented LCO film, the degradation is reduced to 95.71% compared to (100) oriented LCO film, indicating the weakening of the photocatalytic activity. Besides, the photocatalytic activity of (111) oriented LCO film is intermediate between them. Subsequently, the UV–visible absorption spectrum of the methyl orange solution after 3 h of photocatalytic degradation is shown in figure 8. As can be seen, the absorption peak is the strongest when using LCO/(110) LAO as catalyst, while the absorption peak is the weakest with LCO/(100) LAO. These results also suggest that the order of photocatalytic activity of three oriented films is (100) > (111) > (110).

**Figure 9.**
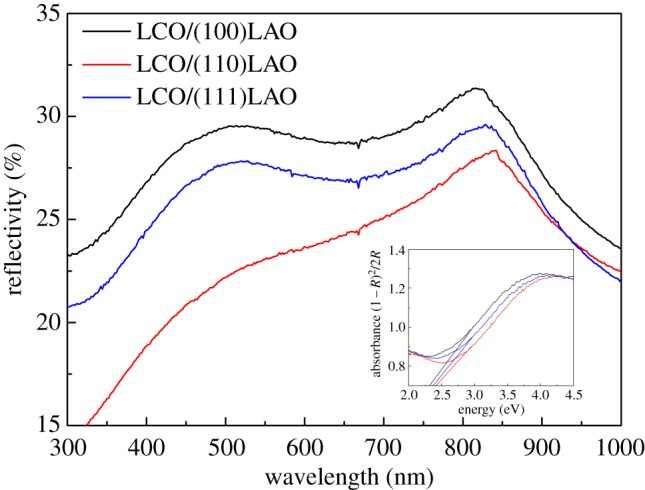
The specular reflectance spectra of LCO epitaxial films on (100), (110) and (111) LAO substrates; the inset shows the corresponding band gap energy calculated by the extrapolation method.

To further investigate the orientation-dependent photocatalytic activity of LCO epitaxial films, the specular reflectance spectra of LCO films were also obtained, as shown in figure 9. It shows that the LCO films on (100) LAO and (111) LAO markedly absorb light in the visible region (500–800 nm), whereas the absorption is much lower for LCO on (110) LAO. This may be an important reason for the observed difference in photocatalytic degradation behaviours and provides a clue for expanding the visible-light-induced photocatalytic application of LCO. From the reflectance spectra of LCO films, the band gap energy (*E*_g_) can be determined by extrapolating the absorption edge onto the energy axis using the linear portion of the straight line fitted curve [*F*(*R*) × *hν*]^2^ versus *hν* (shown in the inset of figure 9), where *F*(*R*) is the Kubelka–Munk function and *hν* is the energy of the incident photon [35]. Here *F*(*R*) = (1 − *R*)^2^/2*R* = *K*/*S*, where *R* is the relative reflectance ratio, *K* the absorption coefficient, and *S* the scattering coefficient. Therefore, the values of *E*_g_ for (100), (110) and (111) oriented LCO films are 2.31, 2.41 and 2.37 eV, respectively. These are all lower than that of bulk LCO (3.02 eV) [36].

It is well known that *E*_g_ can indicate the energy required for electrons to transition from the valence band to the conduction band, which largely reflects the photocatalytic activity of a photocatalyst. For LCO epitaxial films, the photocatalytic activity is mainly determined by the Co^3+^ crystal field splitting energy [4,37]. As is known, LCO has a Co 3*d*^6^ electron configuration. Owing to the CoO_6_ octahedral crystal field, the fivefold 3*d* orbital will split into higher doublet *e*_g_ level and lower triplet *t*_2 g_ level, and the energy difference between them is the crystal field splitting energy (Δ_CF_, approx. 1 eV). Besides, the main contributions to the top of the valence and the bottom of conduction bands are the Co 3*d t*_2 g_ and *e*_g_ orbitals, respectively [38]. Since Δ_CF_ is found to be very sensitive to the variation of the Co–O bond length (*r*), Δ_CF_ ∝ *r*^−5^ [24,30], the strain-induced increase in the mean Co–O bond length (*r*) may reduce Δ_CF_ [10,24]. When irradiated with visible light (approx. 1.5–3.0 eV), photoexcited electrons in LCO can jump from *t*_2 g_ to *e*_g_ level, which is conducive to the photocatalysis. In addition, the binding energy between O and Co ions may also have some influence on photocatalytic activity. The interaction between O and Co will be weakened with the increase in the average Co–O bond length, resulting in a decrease in the binding energy between O and Co ions. This may contribute to the formation of oxygen vacancies on the surface of catalytic materials [10,39] and reduce the electron–hole recombination rate, further promoting photocatalytic reaction.

## 4. Conclusion

We have grown LCO epitaxial films on (100), (110) and (111) LAO substrates, by a simple PAD method. The morphological and structural characteristics show that homogeneous and dense LCO films under biaxial compressive strain in the *ab* plane are all successfully grown epitaxially in accordance with the orientation of LAO substrate. The mean Co–O bond length increases by varying degrees compared with that of bulk LCO, due to the different strain directions of the CoO_6_ octahedron in (100), (110) and (111) oriented LCO films. The largest increase in the mean Co–O bond length exists in LCO film on (100) LAO, while the smallest increase exists in (110) oriented film. The photocatalytic characterization indicates that all the LCO epitaxial films exhibit remarkable photocatalytic performance. The order of photocatalytic activity of the three oriented films is (100) > (111) > (110). It is found that the photocatalytic activity is closely associated with Δ_CF_ and the binding energy between O and Co ions, while the increase in the mean Co–O bond length can lead to the decrease in Δ_CF_ and the binding energy between O and Co ions and further reduce *E*_g_.

## Supplementary Material

Scanning electron microscopy images

## Supplementary Material

X-ray diffraction data

## Supplementary Material

Transmission electron microscopy image

## Supplementary Material

XRD images

## Supplementary Material

UV-visible-near infrared spectroscopy data

## Data accessibility

The datasets supporting this article have been uploaded as part of the electronic supplementary material.
