# Supplementary material for: Orientation-dependent structural and photocatalytic properties of LaCoO3 epitaxial nano-thin films
Source: R Soc Open Sci. 2018 Feb 14;5(2):171376. doi: 10.1098/rsos.171376 (PMC5830743; doi:10.1098/rsos.171376)
Supplement: Scanning electron microscopy images [file rsos171376supp1.doc]

LCO film on (100)LAO





LCO film on (110)LAO





LCO film on (111)LAO
