# Supplementary figures and images for: Orientation-dependent structural and photocatalytic properties of LaCoO3 epitaxial nano-thin films
Source: R Soc Open Sci. 2018 Feb 14;5(2):171376. doi: 10.1098/rsos.171376 (PMC5830743; doi:10.1098/rsos.171376)

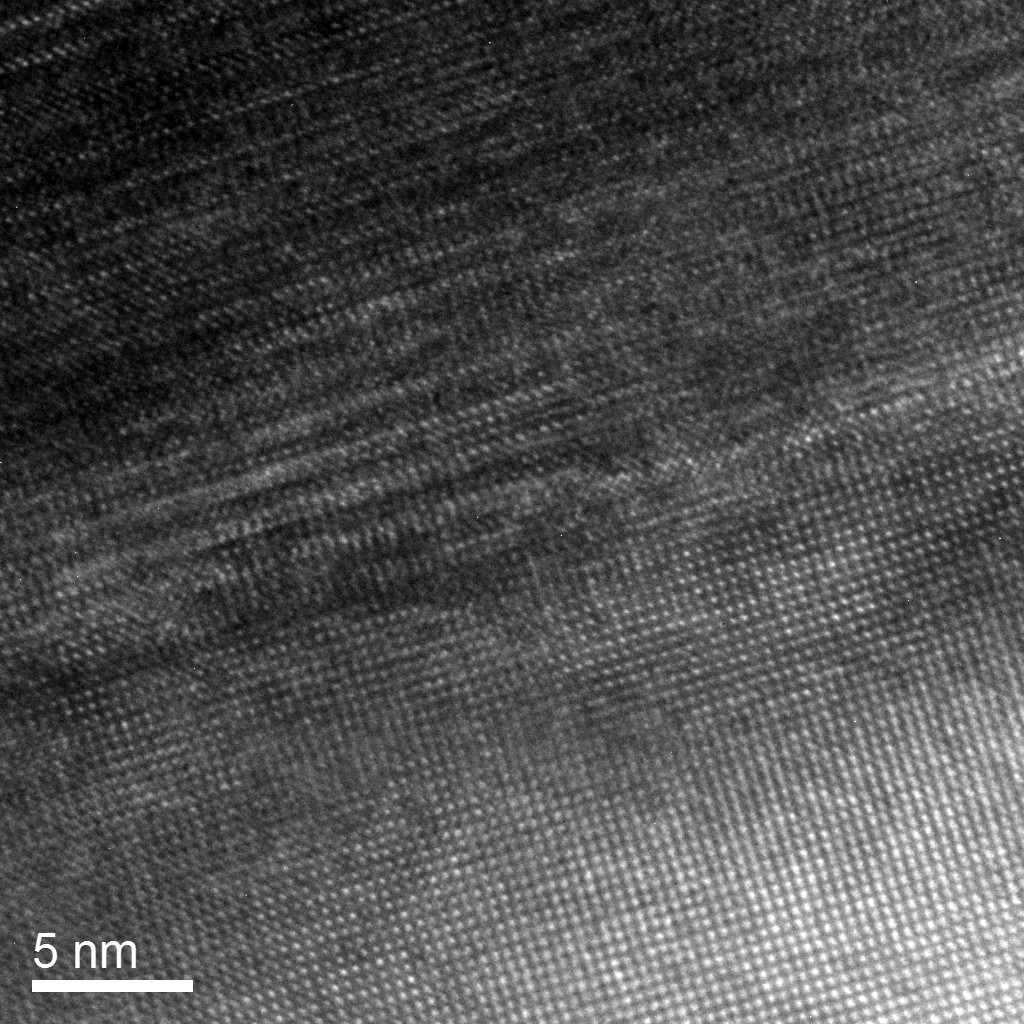


LCO film on (100)LAO


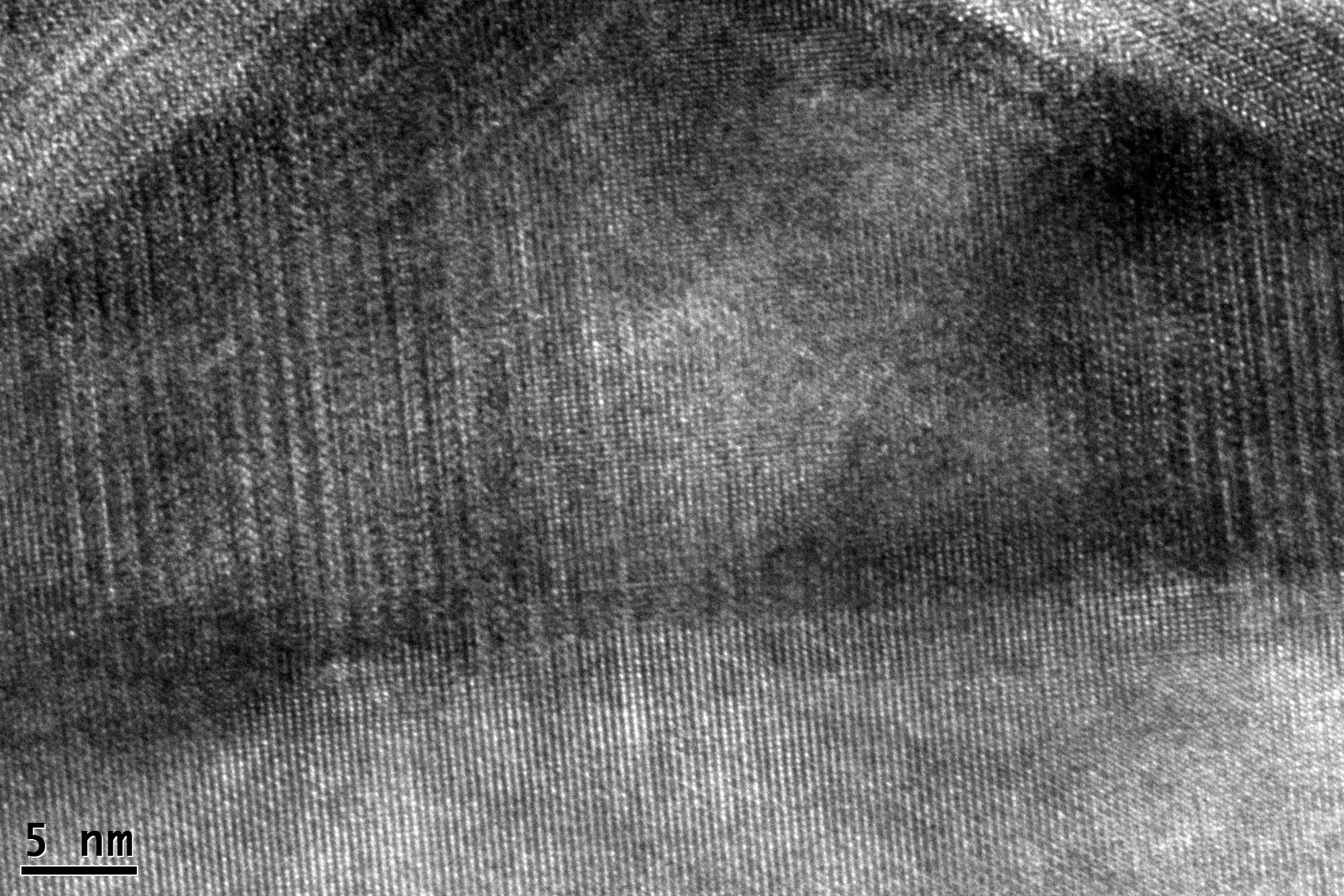


LCO film on (110)LAO


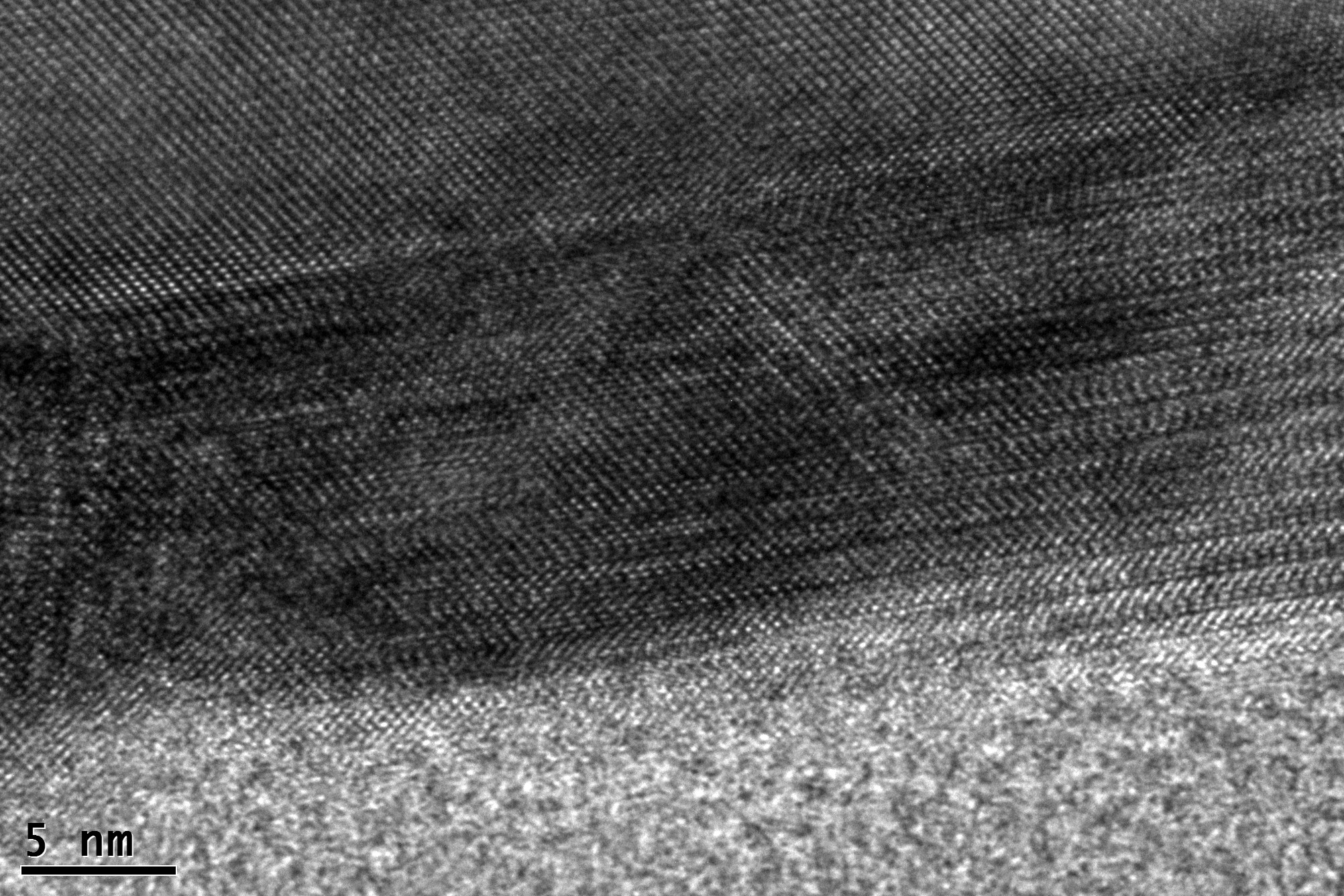


LCO film on (111)LAO

Supplement: Transmission electron microscopy image [file rsos171376supp3.doc]
