# Supplementary material for: Orientation-dependent structural and photocatalytic properties of LaCoO3 epitaxial nano-thin films
Source: R Soc Open Sci. 2018 Feb 14;5(2):171376. doi: 10.1098/rsos.171376 (PMC5830743; doi:10.1098/rsos.171376)
Supplement: XRD images [file rsos171376supp4.doc]

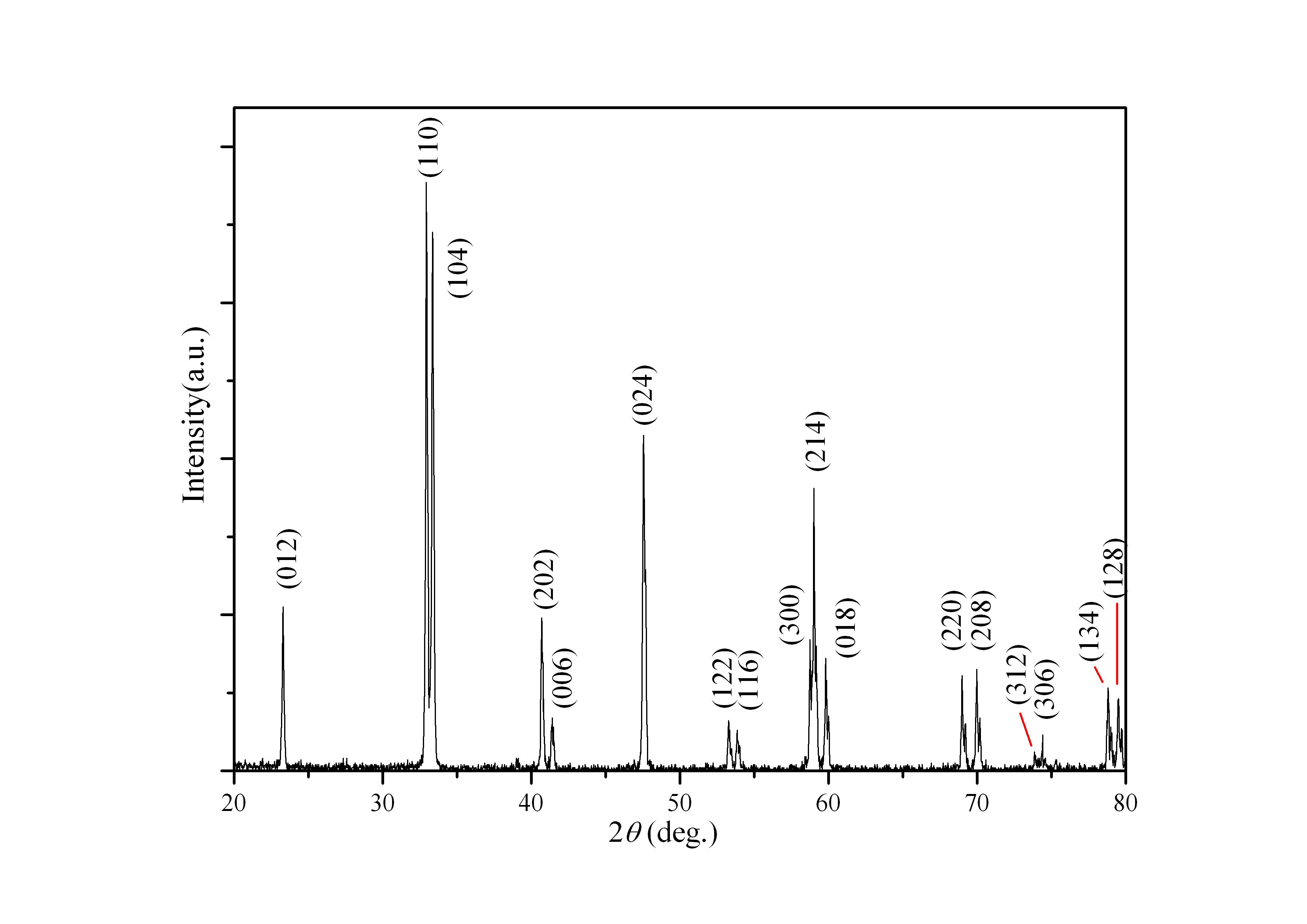

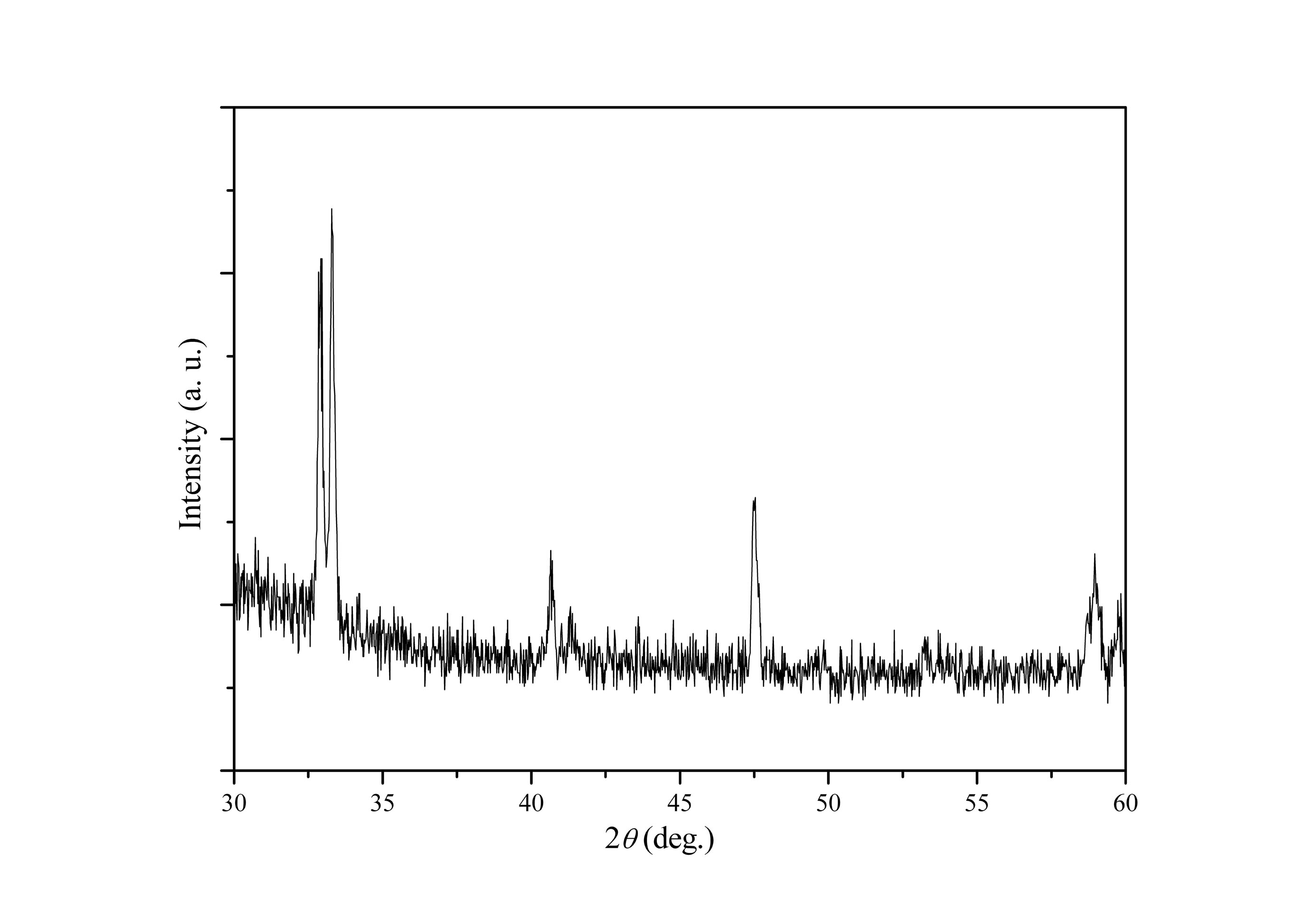


XRD patter of LCO powder. XRD patter of LCO on (100) Si.










The *ω*-scans (rocking-curves) of (100), (110) and (111) oriented

LAO substrates, respectively.
